# Consumption of Dairy Products in Relation to Changes in Anthropometric Variables in Adult Populations: A Systematic Review and Meta-Analysis of Cohort Studies

**DOI:** 10.1371/journal.pone.0157461

**Published:** 2016-06-16

**Authors:** Lukas Schwingshackl, Georg Hoffmann, Carolina Schwedhelm, Tamara Kalle-Uhlmann, Benjamin Missbach, Sven Knüppel, Heiner Boeing

**Affiliations:** 1 Department of Epidemiology, German Institute of Human Nutrition, Nuthetal, Germany; 2 Department of Nutritional Sciences, Faculty of Life Sciences, University of Vienna, Vienna, Austria; University of Catanzaro Magna Graecia, ITALY

## Abstract

**Background:**

The current state of knowledge regarding the association of dairy products and weight gain, overweight, and obesity is based on studies reporting contradicting and inconclusive results. The aim of the present study was thus to clarify the link between dairy consumption in relation to changes in anthropometric measures/adiposity by a meta-analytical approach.

**Methods:**

For the meta-analysis PubMed, EMBASE, Web of Sciences, and google scholar were searched by two independent authors up to May 2016 with no restriction to language or calendar date. Prospective cohort studies reporting about intake of dairy consumption (including milk, yogurt, cheese, butter) and changes in body weight or waist circumference, risk of overweight, obesity, or weight gain were eligible. Pooled effects were calculated using a random effects model, and also a fixed effect model for sensitivity analysis. Due to the heterogeneity of statistical analytical approaches of the studies the analysis were done separately for beta-coefficients of changes in body weight and/or waist circumference per serving of dairy, for differences in weight gain/gain in waist circumference when comparing extreme categories of dairy consumption, and for odds ratios in regard to weight gain, overweight/obesity, or abdominal obesity.

**Findings:**

24 studies (27 reports) met the inclusion criteria for the systematic review, and 22 studies provided sufficient data for inclusion in the meta-analysis. The meta-analysis of the five studies on changes in body weight per serving of dairy no significant results could be found for whole fat dairy and low fat dairy. However, there was inverse association between changes in body weight for each serving’s increase of yogurt (beta: -40.99 gram/year, 95% CI, -48.09 to -33.88), whereas each serving’s increase of cheese was positively associated (beta: -10.97 gram/year, 95% CI, 2.86 to 19.07). Furthermore, the highest dairy intake category was associated with a reduced risk of abdominal obesity (OR: 0.85; 95% CI, 0.76 to 0.95), and risk of overweight (OR: 0.87; 95% CI, 0.76 to 1.00) compared to the lowest intake category. No significant association could be observed for risk of weight gain.

**Conclusion:**

In summary the results of the meta-analysis still reflect that dairy consumption was not positively related to changes in body weight. Yogurt was the only dairy food that showed some evidence for a beneficial effect, where higher intakes were inversely associated a reduced risk of obesity, changes in body weight or waist circumference. Further research is needed, since the overall interpretation of the results is limited by heterogeneous risk estimates.

## Introduction

Most dietary guidelines recommend the consumption of milk and dairy products as important components of a healthy, well-balanced diet. Dairy products are a high-quality protein source providing a substantial proportion of the recommended adult nutrient intake of calcium, iodine, riboflavin, and vitamin B_12_. It is thus well reasoned that for instance public health measures in the USA recommend an increased intake of fat-free or low-fat milk and milk products [1].

Several recent meta-analyses of prospective cohort studies have shown that dairy consumption is associated with a reduced risk of cardiovascular disease, hypertension, stroke, and diabetes [2–5]. In line with these findings, a recently published meta-analysis of 76 studies including 659,298 participants showed that higher levels of circulating margaric acid (a component of ruminant milk fat) was associated with a 23% risk reduction of coronary heart disease [6].

While some meta-analyses have highlighted more favorable effects of low-fat dairy compared to higher-fat dairy products [4, 7], it has been demonstrated that study participants who consumed more dairy fat and/or high-fat dairy foods at baseline, were leaner and/or gained less weight over time compared to participants who consumed less [8]. In contrast, a current meta-analysis of clinical trials could not support the beneficial effect of an increased dairy consumption on body weight and fat loss in long-term studies or studies without energy restriction [9]. While another meta-analysis of randomized controlled trials (RCTs) has shown that increasing both, whole and low fat dairy food consumption increases weight [10], a number of short-term or energy-restricted RCTs report modest benefits of dairy products in facilitating weight loss [9]. A recent summary of RCTs showed that dairy intake resulted in greater fat mass reduction in the presence of energy restriction in the short-term compared to a control group [11].

As a result of the available contradicting and inconclusive results, the association of dairy products and weight gain, overweight, and obesity has been critically questioned [12, 13]. This regards the biological nature (e.g. modulation of adipocyte lipid metabolism) as well as the overall quantitative effect size of the relationship in free living populations. Since we could not find data on overall effect size of dairy consumption on anthropometric changes, including changes in body weight and waist circumference (WC), and risk of weight gain/overweight/obesity we conducted a quantitative meta-analysis of cohort studies with the focus on dairy consumption and its association with adiposity as complement to the already conducted meta-analyses of RCTs.

## Materials and Methods

This systematic review was planned and conducted according to the standards of the Meta-analysis of Observational Studies in Epidemiology [14]. Our protocol has been registered in the PROSPERO International Prospective Register of Systematic Reviews. (Registration number: CRD42014013997)

### Literature search

A literature search was performed in PubMed, EMBASE, Web of Science, and google scholar (until May 2016) with no restrictions to language, and calendar date using the following search terms: *(“dairy” OR " milk" OR " yogurt" OR " cheese") AND (" weight" OR " waist circumference" OR “BMI” OR “body mass index” OR " obesity" OR “hip”) AND (" longitudinal" OR " prospective" OR " cohort" OR " change" OR “follow-up")*. Moreover, the reference lists from retrieved articles and systematic reviews, were checked to search for further relevant studies as well as citations of those articles. Literature search was conducted independently by two authors (LS and TKU), with disagreements resolved by consensus.

### Eligibility criteria

Studies were included in the meta-analysis if they met all of the following criteria: *(i)* longitudinal study design; *(ii)* data related to consumption (quantitative amount; i.e. servings per day/week, gram per day/week intake) of dairy products (including milk, yogurt, cheese, butter); the primary outcomes were: changes in body weight, either measured continuously (g/year) or binary as incident major weight gain (e.g. incidence of gaining a specific amount of weight, risk of overweight, abdominal obesity); the secondary outcomes were: changes in waist circumference; *(iii)* reported adjusted beta-coefficients with corresponding standard error or data necessary to calculate these (95% confidence intervals, (95% CI), standard deviations, p-values); *(iv)* mean differences with corresponding standard error in change in measure of adiposity over time between participants with the highest and lowest intake category; *(v)* adjusted odds/risk/hazard ratios with corresponding 95% CI or standard error comparing the highest vs. lowest dairy intake category; *(vi)* when a study appeared to have been published in duplicate, the version containing the longest follow-up was selected; *(vii)* study participants were free of chronic disease (cardiovascular disease, cancer) at baseline of study; *(viii)* only adults were included (minimum of 18 years of age).

### Data extraction and quality assessment

We extracted the following data from each study: first author’s name, publication year, location, cohort name, type of outcome, population (age, sex, and sample size), follow-up duration, dietary assessment method, definition of dairy products (including the unit of consumption), whether dairy was modeled continuously or categorical, outcome definition, outcome assessment, statistical analysis method, and variables adjusted for. Further, we extracted data of the effect sizes with their corresponding uncertainties. When a study reported only separate effect estimates for males and females, they were treated as different studies. When a study provided effect estimates for different degrees of adjustment, those from the most completely adjusted model were chosen. Study quality was assessed in accordance with a recent meta-analysis of sugar intake and adiposity [15], and included risk of bias (selection of exposed and unexposed in cohort studies from different populations; partially flawed measurement of both exposure (i.e. relied only baseline dairy consumption; measurement error) and outcome (i.e. self-reported); Inconsistency: i.e. point estimates vary widely across studies; confidence intervals shows minimal or no overlaps; statistical test for heterogeneity shows a low p-value; I^2^ is large [16, 17].

### Statistical analysis

We classified the prospective studies according to the type of measure of association they used [18]:

1. Adjusted beta-coefficients for the association of dairy intake with subsequent changes in anthropometric outcomes per g intake;
2. Mean differences of change in anthropometric measures over time comparing the highest and lowest category of dairy consumption;
3. Odds/risk/hazard ratios for risk of overweight or abdominal obesity, or for risk of gaining a particular amount of weight.

Study specific results were pooled with random effects models using the DerSimonian-Laird method, which incorporated both within and between study variability [19]. To evaluate the weighting of each study, the standard error of the beta-coefficient, mean differences, and for the logarithm odds/risk/hazard ratios of each study was calculated and regarded as the estimated variance of the beta-coefficients, mean differences, and odds ratio using the DerSimonian-Laird method and the inverse variance method [19]. Mean changes in body weight and waist circumference and odds/risk/hazard ratios of incident weight gain, overweight, abdominal obesity were pooled comparing participants who had the highest intakes of dairy with those who had the lowest intakes.

Separate meta-analyses were performed for the beta-coefficients per g intake, for the mean changes and for the odds/risk/hazard ratios (adiposity measures) taking the different categories of intake of dairy products such as total dairy, whole fat dairy, low-fat dairy, cheese, yogurt, milk). Heterogeneity was tested with the Q statistic and quantified with the I^2^ statistic. I^2^ values >50% were indicative for substantial heterogeneity across studies [20]. Funnel plots, in which the effect sizes are plotted against their corresponding uncertainty, were used to assess potential publication bias if at least 10 studies were available [21]. In order to check sensitivity of the results we also conducted all analysis with a fixed effects model using an inverse variance method [19]. We used the “metan” command in Stata 12.0 (Stata Corp. 2011, Texas, USA) for all meta-analyses.

## Results

### Literature search and study characteristics

24 studies (27 reports) met the inclusion criteria for the systematic review [22–48], and 22 studies provided sufficient data for inclusion in the meta-analysis (reported quantitative amount of dairy consumption) [23–44, 46–48] (S1 PRISMA Checklist). The study selection process is reported in the Flow diagram (S1 Fig and S1 Table). Of these, seven studies (eight reports) analyzed changes in body weight solely [27–30, 33, 37, 45, 48], four studies (five reports) changes in waist circumference solely [25, 34, 35, 43, 44] and five studies both changes in body weight and waist circumference [32, 38, 40–42], while ten studies (11 reports) measured changes in risk of weight gain, overweight, and abdominal obesity [23, 24, 26, 27, 31, 36, 39, 44, 46–48]. General study and specific characteristics are given in **Table 1**. Sample size varied between 76 and 120,077 participants with a follow-up time ranging from 9 months to 23 years. All studies except for three (Iran, Korea, China) [24, 39, 43] were conducted in North America and Europe. The dairy (grams/servings/units, percentage of energy intake, frequency of consumption, or tertiles/quartiles/quintiles) categories and estimates (beta-coefficients, mean changes, odds/risk ratios) were heterogeneous. All but four studies, used food frequency questionnaires as dietary assessment method [22, 26, 32, 38].

**Table 1:** Characteristics of prospective observational studies included in the qualitative systematic review or quantitative meta-analysis.

Author, year	Country	Cohort	Outcome	Population, Follow-up (years)	Age at entry (years)	Sex	Dairy products (categories)	Adjustment	Dietary assessment
Babio et al. 2015	Spain	PREDIMED	Risk of abdominal obesity	1868, 3.2	55–80	M&W	Tertiles of yogurt consumption (≥450g/daily vs. ≤287g/daily) (low fat vs. high-fat);	Intervention group, sex, age, leisure time physical activity, BMI, current smoker, former smoker, use of hypoglycemic, hypolipidemic, antihypertensive, and insulin treatment at baseline, plus mean consumption during follow-up of vegetables, fruit, legumes, cereals, fish, red meat, cookies, olive oil, and nuts, as well as alcohol	FFQ
Drapeau et al. 2004	Canada	Quebec Family Study	Changes in body weight/ WC	248, 6	18–65	M&W	Skimmed and partly skimmed milk (BW, WC), Yogurt with <2% fat (WC)	All variables are adjusted for initial age, baseline body-weight, or adiposity indicators and changes in daily physical activity level (only yogurt)	3-day dietary records
Duffey et al. 2010	USA	CARDIA	Risk of high WC (>102 cm for men, >88 cm for women)	5040, 20	18–30	M&W	Low fat milk, whole fat milk	For race, sex, exam center, year 0 age, weight, smoking status, energy from food, total physical activity, energy from the 3 other beverages, energy from alcohol	FFQ
Esfahani et al. 2014	Iran	Teheran Lipid and Glucose Study	Changes in body weight (≥0.5 kg)	851, 3	19–78	M&W	Dairy foods (increased or decreased intake)	Age, body weight, education level, smoking behavior, physical activity	FFQ
Fumeron et al. 2011	France	DESIR	Changes in WC	3417, 9	30–65	M&W	Dairy consumption score (4 vs 1)	Gender, age, smoking, physical activity, fat intake, BMI	FFQ
Funtikova et al. 2015	Spain	Population based survey Girona	Changes in WC, Risk of abdominal obesity	2181, 10	25–74	M&W	100 kcal increase in whole milk, or skim and low-fat milk, Highest (≥200 ml) vs. lowest (no consumption) tertile of whole milk, skim and low-fat milk	Sex, age, baseline WC, smoking, energy intake, education level, physical activity, energy-under and over-reporting. Modified Mediterranean diet score	FFQ
Holmberg et al. 2013	Sweden	Cohort from the Swedish National Farm Register	Central obesity (WHR≥1)	1322, 23	40–60	M&W	High fat versus low fat milk, Butter versus low fat margarine/nothing, Whipping cream vs never	Crude	15-item questionnaire
Kaikkonen et al. 2015	Finland	Young Finns Study	Changes in weight, and BMI (baseline)	1715, 6	24–39	M&W	Monthly use of industrial milk products	n.d	FFQ
Martinez-Gonzalez et al. 2014; Sayón-Orea et al. 2015	Spain	SUN	Changes in body weight; and risk of overweight and obesity; risk of abdominal obesity	8516, 6.6	Mean age 37.1 (±10.8)	M&W	Quintiles of yogurt consumption (low fat vs. high-fat) (0–2/week vs. >7/week)	Sex, age, baseline weight, physical activity, hours of TV watching, hours spent sitting down, smoking status, snacking between meals, following a special diet, total energy intake, adherence to the Mediterranean diet, marital status, and years of education	FFQ
Mozaffarian et al. 2011; Pan et al. 2013	USA	NHS I, NHS II, HP	Changes in body weight (kg/each 4 years)	50422, 20; 47898, 12; 22557, 20	52.2±7.2, 37.5±4.1, 50.8±7.5	M&W	Whole fat dairy (butter, cheese, milk); low fat dairy (yogurt, milk)	Age, BMI, television watching, sleep duration, physical activity, alcohol use, smoking, and all of the dietary factors	FFQ
Nikolaou et al. 2014	United Kingdom	students	Changes in body weight	1275, 9-months	20	M&W	2–3x daily 30grams vs. lower intake	Baseline weight, height, age, and gender	FFQ
Pereira et al. 2002	USA	Cardia	Obesity (among individuals overweight at baseline)	3157, 10	18–30	M&W	All dairy products Reduced fat; High fat; Milk and milk drinks; Cheese and sour cream; Butter and cream; Dairy-based desserts; yogurt	Age, sex, race, calorie intake, study center, baseline BMI, education, daily alcohol intake, smoking status, physical activity, vitamin supplements, dietary factors, dairy components	FFQ
Poddar et al. 2009	USA	College students	Changes in body weight, WC	76, 1	Mean age = 19.2 ±0.2	M&W	Low fat dairy (0.14 vs. 0.84 servings/day)	Energy intake, race, sex, BMI, percent total body fat	7-day food record
Rajpathak et al. 2006	USA	HP	Changes in body weight	19615, 12	40–75	M	Quintiles of Total dairy, High fat dairy, Low fat dairy	Age, baseline weight, change in smoking status, baseline and change in physical activity; changes in intake of calories, alcohol, total fat, cereal fiber, glycemic load, fruit and vegetables, whole grains, *trans* fat, caffeine, and low- and high-caloric soft drinks; and baseline intakes of all dietary covariates	FFQ
Rautiainen et al. 2016	USA	WHS	Changes in body weight; Risk of overweight or obesity	18438, 11.2	≥45	W	Quintile of Total dairy, High fat dairy, Low fat dairy	Age, randomization treatment, smoking status, physical activity, postmenopausal status, postmenopausal hormone use, history of hypercholesterolemia, history of hypertension, multivitamin use, alcohol intake, energy intake, fruit and vegetable intake, BMI	FFQ
Romaguera et al. 2011; Halkjæret al. 2009	European Union	EPIC	Changes in WC	48631, 5.5	Exclusion baseline >60 years; And follow-up >65	M&W	Dairy products, Milk, Yogurt, Cheese (100 kcal increments in intake)	Total energy intake, age, baseline weight, baseline height, baseline WC, BMI, smoking, alcohol intake, physical activity, education, follow-up duration, menopausal status (women only), and hormone replacement therapy use (women only)	FFQ
Rosell et al. 2006	Sweden	Swedish Mammography Cohort	Weight gain (≥1 kg)	19352, 8.8	40–55	M&W	Whole milk and sour milk, 3% fat, Medium-fat milk, 1.5% fat, Low-fat milk and sour milk, 0.5% fat, Cheese, Butter	Age, height and weight at baseline, education, parity, intake of energy, protein, fat, carbohydrates, fiber, alcohol and changes in intakes during follow-up, type of dairy	FFQ
Sanchez-Villegas et al. 2006	Spain	SUN	Changes in body weight	6319, 2	University graduates Mean: 38	M&W	Whole fat dairy: <122.4, 122.4–254.2, > 254.2	Age, gender, baseline BMI, smoking, physical activity, alcohol consumption, energy intake, change in dietary habits	FFQ
Samara et al. 2013	France	STANISLAS	Changes in body weight, WC	588, 5	28–60	M&W	Milk, yogurt and cottage cheese, cheese	Age, physical activity, alcohol, cigarette consumption, energy intake, education level, mean adequacy ratio index, value of MetS-related variable at entry	3-day food diary
Shin et al. 2013	Korea	Anseong and Ansan cohort of the Korean Genome and Epidemiology Study	Risk of abdominal obesity	7240, 3.8	40–69	M&W	Dairy and milk consumption (none vs. ≥ times/week)	Age, sex, physical activity, daily alcohol consumption, smoking pack-year, income, education, total energy intake	FFQ
Snijder et al. 2008	Holland	Hoorn Study	Changes in body weight, and WC	1124, 6.4	50–75	M&W	Total Dairy (servings/day)	Age, sex, total energy intake, baseline value of the outcome variable, alcohol intake, smoking, physical activity	FFQ
Vergnaud et al. 2008	European Union	SU.VI.MAX	Changes in body weight, and WC	2267, 6	25–70	M&W	Total dairy, milk, cheese, yogurt (Quartile consumption)	Intervention group, baseline value of the outcome, educational level, smoking status, physical activity level, energy intakes, mean adequacy ratio, and intakes of alcohol	FFQ
Wang et al. 2013	USA	Framingham Heart Study Offspring	Changes in body weight, and WC	3440, 12.9	28–62	M&W	total dairy; high-fat; low-fat; skim low fat-milk; cheese; yogurt (<1 servings/1-<3 servings, ≥3 servings)	Sex and time-varying variables including age, smoking status, physical activity and weight at the beginning of each exam interval, and average total energy intake and Dietary Guidelines Adherence Index (DGAI) score during each exam interval	FFQ
Zong et al. 2013	China	Nutrition and Health of Aging Population in China	Changes in WC	3289, 6	50–70	M&W	Serving dairy	Age, sex, region, residence, smoking status, family history of diabetes, BMI (not for BMI and waist circumference), dietary fiber intake, and baseline values of respective variables	74-item FFQ

BMI: body mass index; FFQ: food frequency questionnaire; MUFA: monounsaturated fatty acids; n.d., no data; WHR: waist-to-hip ratio.

### Changes in body weight (beta coefficients and mean differences)

**Fig 1:**
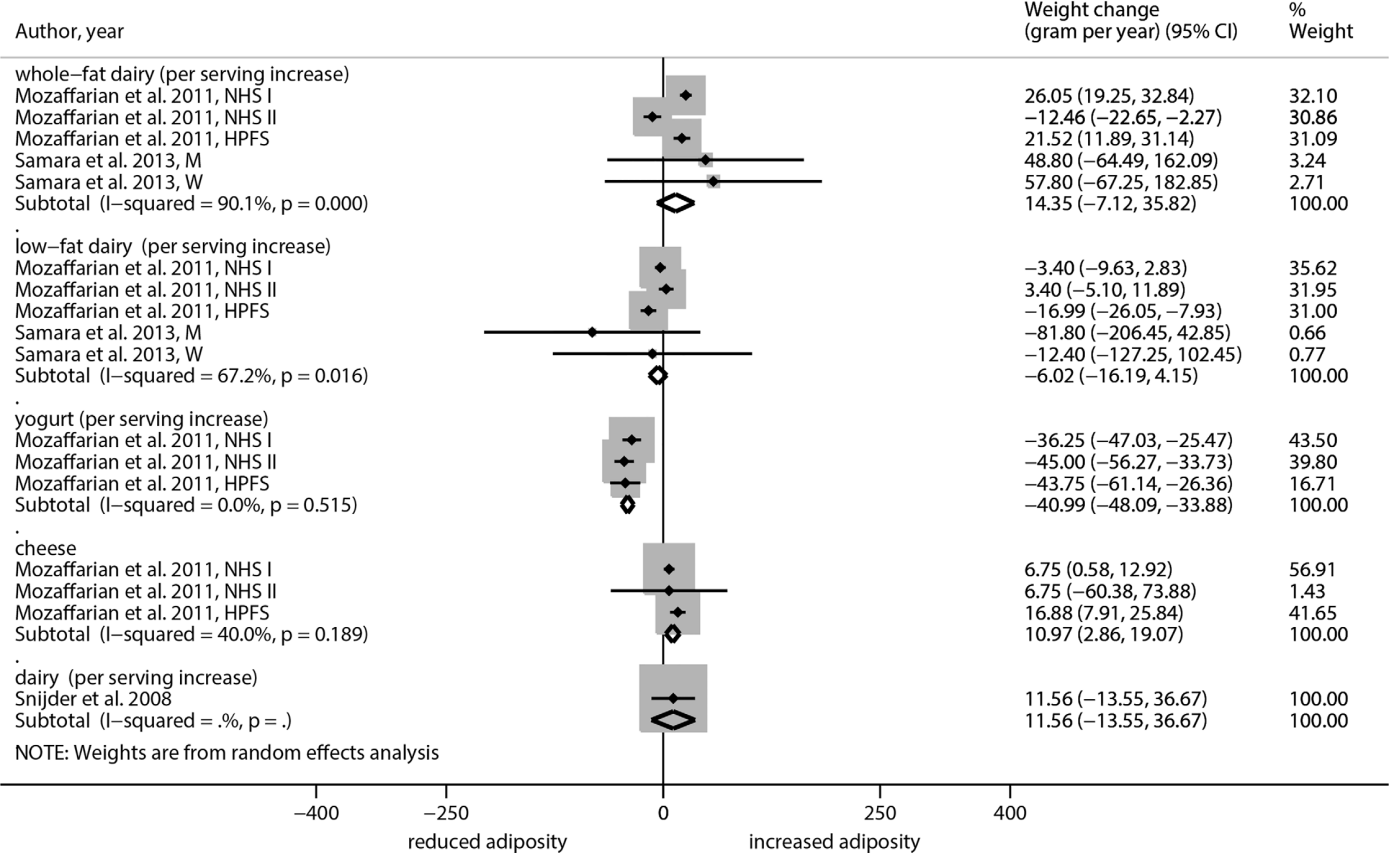
Forest plot of associations between changes in body weight (gram/year) and dairy consumption in cohort studies of adults.

In studies that analyzed the association between whole fat dairy intake and changes in body weight, one study found a significant positive association. In this study by Mozaffarian et al. [28] every serving increase was associated with a 26.05 gram/year (95% CI, 19.25 to 32.84) increase among the Nurses’ Health Study I cohort and a 21.52 gram/year (95% CI, 11.89 to 31.14) increase among the participants of the Health Professional Follow-up Study. In the pooled analysis of studies that analyzed the association between whole fat dairy intake and changes in body weight, it could be shown that every serving increase was associated with a non-significant 14.35 gram/year (95% CI, -7.12, 35.82; I^2^ = 90.1%) annual weight increase. For low fat dairy, a non-significant inverse association could be observed with a -6.02 gram/year (95% CI, -16.19 to 4.15; I^2^ = 67.2%) annual weight decrease per serving increase in the random effects model (Fig 1). Surprisingly, the fixed effect models showed significant results in both cases when the sensitivity analysis was performed. On the other side, an inverse association between changes in body weight for each serving’s increase of yogurt (beta: -40.99 gram/year, 95% CI, -48.09 to -33.88) could be observed, whereas each serving’s increase of cheese was positively associated with changes in body weight (beta: -10.97 gram/year, 95% CI, 2.86 to 19.07).

No significant effects on changes in body weight could be observed when pooling the highest vs. lowest dairy, yogurt, cheese, whole-fat dairy, and low-fat dairy intake categories (S2 Fig).

### Changes in waist circumference (beta coefficients and mean differences)

Concerning changes in waist circumference, the study by Romaguera [34] observed that every 100 kcal increase in milk, yogurt, or cheese was associated with a decrease in waist circumference in both men and women, whereas no significant association between a 100 kcal increase in whole, and skim/low fat milk could be observed. In the random effects model the value for milk was -0.02 cm/year (95% CI, -0.02 to -0.01; I^2^ = 47.9%) annual waist circumference change per serving increase, for yogurt the value was -0.02 cm/year (95% CI, -0.04 to -0.01; I^2^ = 0%), and for cheese -0.02 cm/year (95% CI, -0.04 to -0.00; I^2^ = 0%) (S3 Fig).

When comparing the highest versus lowest categories of dairy consumption and changes in waist circumference, it could be shown that dairy was associated with a non-significant (-0.07 cm/year, 95% CI, -0.09 to 0.01; I^2^ = 54%) annual decrease in the random effects model (S4 Fig). No significant associations could be observed for milk, low-fat dairy, high-fat dairy, yogurt and cheese consumption.

### Risk of overweight, abdominal obesity/weight gain (odds/risk/hazard ratio)

**Fig 2:**
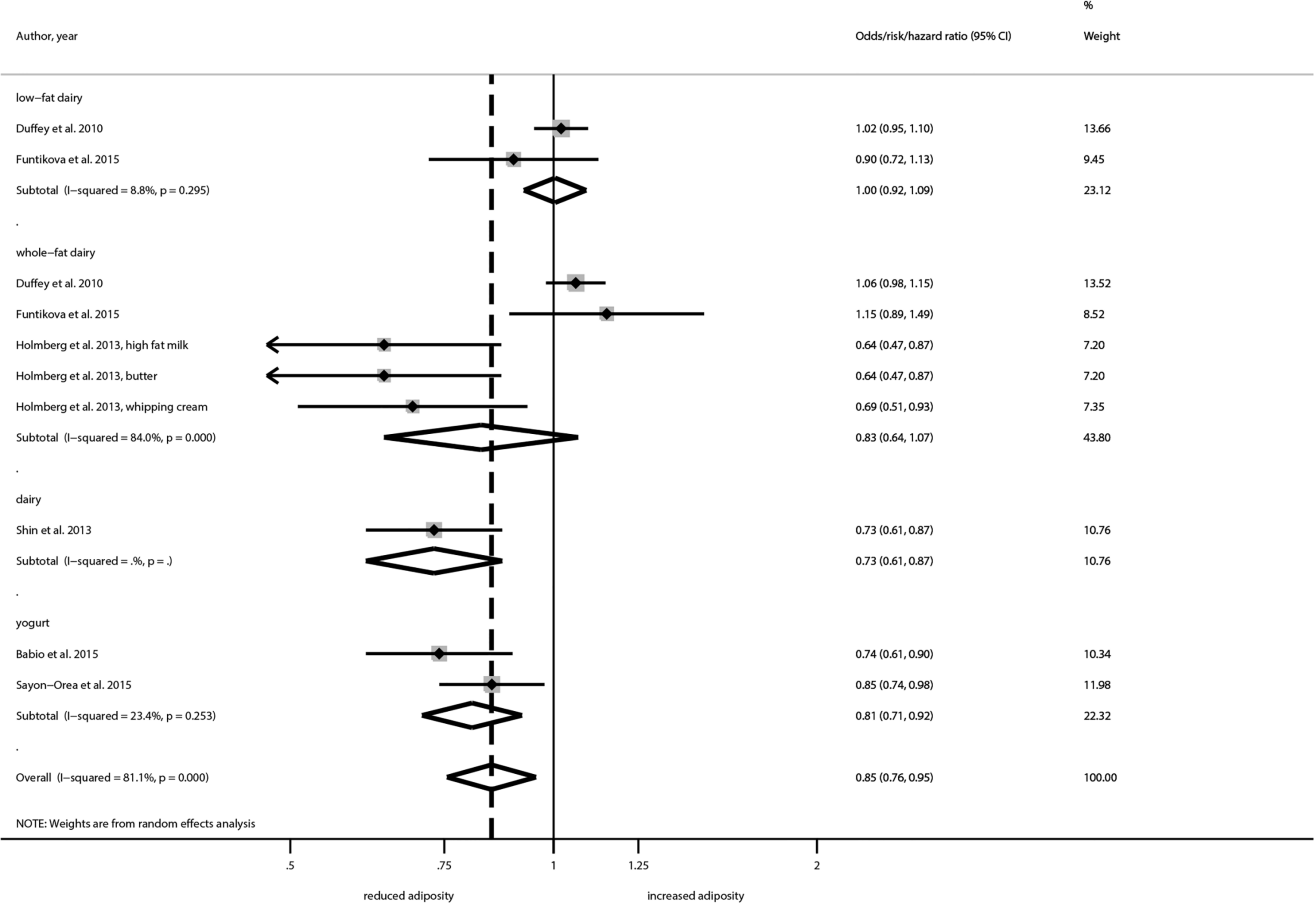
Forest plot showing pooled OR/RR/HR with 95% CI for abdominal obesity comparing categories of milk intakes.

Pooling three studies [27, 31, 48] showed a reduced risk between dairy consumption and risk of overweight (OR/RR/HR: 0.87; 95% CI, 0.76 to 1.00; I^2^ = 0%), one of which focused on yogurt consumption (S5 Fig). A significant inverse association could be also observed between dairy products and risk of abdominal obesity (OR/RR/HR: 0.85; 95% CI, 0.76 to 0.95; I^2^ = 81.1%) (Fig 2). No significant associations were observed for overall dairy intake and weight gain, and the intake of milk and risk of weight gain and overweight. However, sensitivity analysis comparing low-fat vs. whole-fat dairy yielded in a significant reduction for adiposity only for whole fat dairy products (S6 Fig).

### Study quality, methodological issues

The present systematic review included 24 longitudinal studies (27 reports) lasting at least 9 months, and in which data relating to an association between dairy products and a measure of adiposity could be extracted. Of these 24 studies, five used self-reported estimates of body weight or waist circumference, validated in a subsample [27, 28, 30, 33, 36, 48] and three used self-assessed anthropometric parameters only in the follow-up [34, 37]. Regarding the dietary assessment methods, all but four applied food frequency questionnaires, two of which used dietary records [22, 32], one repeated 24-h dietary records [41], and one study a 15-item questionnaire [26]. Collected exposure data from questionnaires where the validity for assessing dairy intakes was not stated or not assessed included five studies [26, 32, 38, 40, 45], and 13 (14 reports) out of 23 studies provided estimates that were adjusted for total energy intake. The wide range of methods of assessing dairy exposures and adiposity outcomes as well as the inconsistency in the covariates used to adjust analyses made pooling studies difficult.

## Discussion

Overall the results of the present meta-analysis showed that higher intakes of dairy products were not associated with increased body weight gain. In the fixed effects model whole fat dairy products were associated with a small but significant increase in body weight, whereas low-fat dairy was inversely related to changes in body weight. An increase in dairy intake was inversely related to changes in waist circumference. The pooled results from the Harvard cohorts suggest that higher yogurt intake is the only dairy food inversely related with changes in body weight [28], whereas each serving’s increase of cheese was positively associated with body weight. Furthermore the comparing the highest category of dairy consumption was associated with a reduced risk of overweight, and abdominal obesity (mainly contributed throughout whole-fat dairy products).

As stated by Prentice [49], there are only two foods consumed by humans that have been designed to meet the entire nutrient needs of a complex organism: milk and eggs. Diets containing high proportions of both foods are thus unsurprisingly beneficial for healthy growth and development.

To summarize the relationship between dietary calcium intake and BMI after correction for trials effect, a mixed model regression analysis was conducted using mean data reported in the analyzed studies. While dairy products remain the major source for calcium [50], the role of dairy components in the regulation of energy metabolism, weight and body fat regulation, has not been clearly elucidated. However, the most reasonable mechanism refers to a modulation of adipocyte lipid metabolism and fatty acid absorption from the gastrointestinal tract by the effects of dietary calcium on intracellular calcium [51]. Additionally, research indicates that high calcium intake may reduce lipogenesis and increase lipolysis by hormone regulation [52]. A meta-analysis of clinical trials showed that calcium supplementation was associated with a significant weight and fat mass reduction. However, this meta-analysis is not interpretable and has major methodological flaws, since the authors used the standard error instead of the standard deviation for one study, causing an overestimation of the effect size [53].

In addition, other dairy compounds with beneficial effects on weight and body fat loss have been proposed. As such, whey protein (effects on muscle sparing and lipid metabolism [54–56]), conjugated linoleic acid (regulation of adipogenesis, inflammation, and lipid metabolism [57]) are promising constituents. Additional to their effect on satiety [58], milk proteins are insulinotropic. Taken together, milk proteins may play an important role in explaining the association between dairy consumption and body weights [59].

Evidence from the present meta-analysis indicates an inverse association between yogurt consumption and weight gain. Nutrients from yogurt have a higher bioavailability compared to other forms of dairy [60]. Recent research indicates that gut microbiota plays a decisive role in weight control [61–63]. It is hypothesized that probiotic yogurt enhances the growth of beneficial intestinal microbiota and modulates gut function through regulation of the immune system. These effects have been discussed to facilitate weight loss or weight maintenance [64], which is also the crucial factor in the prevention of type 2 diabetes mellitus [65]. In line with our results, meta-analyses of prospective studies have shown that fermented dairy products were associated with a 12–17% reduction in type 2 diabetes mellitus [4, 5].

To our knowledge, no high quality long-term RCTs analyzing weight outcome were conducted, so the level of evidence is limited.

When analyzing dairy products as part of a healthy diet, the unfavourable effects cannot be left disregarded. First, according to data from the NHANES III certain dairy products (regular cheese, dairy desserts, low-fat milk, whole-fat milk, and butter) contribute to 25% of the saturated fat intake in the US diet [66]. In European countries, these numbers are even higher with a proportion of 41% [67]. Second, reducing saturated fat intake is pivotal of most dietary recommendations to reduce the risk of coronary heart disease [68, 69].

A number of prospective epidemiological studies have investigated the dairy-weight loss hypothesis [23–44]. It should be noted that US and European prospective cohort studies are susceptible to unmeasured confounding by individual dietary components. Diets arise from a complex network of foods consumed within and between meals where clustering of “healthy” or “unhealthy” behaviors is common. Specifically, yogurt and milk consumption have often been associated with a better overall dietary profile and inversely associated with the consumption of sugar-sweetened beverages, especially soda and fruit juices [70–72].

Regarding anthropometric outcomes, a longitudinal study showed that a healthy dietary pattern high in reduced-fat dairy was inversely associated with BMI and with waist circumference [73]. In the case of dietary patterns as exposures, the individual contribution of specific food groups to the effect cannot be quantified, but rather the results are seen as the effect of the interaction of this complex network of foods over the outcome.

Dairy consumption patterns may differ across countries. As such, in the US, a large proportion of dairy fat is consumed via commercial foods such as ice cream and pizza [74]. Although this holds true throughout the industrialized world, there is a stronger tradition in European countries to consume whole-fat dairy products (plain cheeses, unsweetened yogurt and plain butter).

### Limitations

The present systematic review has several limitations. First, baseline and follow-up body weight assessment were measured differently across cohorts (data based on self-report and exact weight measure method). For some included studies, only baseline dairy consumption was used (assuming a stable consumption over time). Common problems in prospective cohort studies, like measurement errors, have to be noted. In most studies, dietary intake is calculated from self-assessment tools such as FFQs [75]. As it is difficult to disentangle the effects of dairy fat from the food within which it was consumed using observational methods, this may also be considered as an additional confounding factor [8].

Second, the included studies showed substantial heterogeneity with respect to the analyzed population size, follow-up length, baseline age, dairy categories, adjustment factors, and outcome estimates.

Thirdly, since the power of each meta-analysis was limited by the low number of studies, potential confounding explored by stratified analysis could not be conducted.

## Conclusion

In conclusion, the results of the present meta-analysis indicate that dairy consumption was not related to changes in body weight, but inversely associated to changes in waist circumference, risk of overweight and abdominal obesity. Yogurt was the only dairy food that showed some evidence for a beneficial effect, where higher intakes were inversely associated with a reduced risk of obesity, changes in body weight and waist circumference. Further research is needed, since the overall interpretation of the results is limited by heterogeneous risk estimates.

## Supporting Information

S1 PRISMA Checklist (DOC)

S1 Fig Flow diagram. (DOCX)

S2 Fig Forest plot of mean changes in body weight (gram/year) comparing the highest vs lowest dairy consumption category. (EPS)

S3 Fig Forest plot of associations between changes in waist circumference (cm/year) and dairy consumption in cohort studies of adults. (EPS)

S4 Fig Forest plot of mean changes in waist circumference (cm/year) comparing the highest vs lowest dairy consumption category. (EPS)

S5 Fig Forest plot showing pooled OR/RR/HR with 95% CI for overweight comparing categories of dairy intakes. (EPS)

S6 Fig Forest plot showing pooled OR/RR/HR with 95% CI for adiposity comparing low vs. whole−fat dairy intakes. (EPS)

S1 Table Full-text articles excluded with reasons. (DOCX)
