# Supplementary material for: Consumption of Dairy Products in Relation to Changes in Anthropometric Variables in Adult Populations: A Systematic Review and Meta-Analysis of Cohort Studies
Source: PLoS One. 2016 Jun 16;11(6):e0157461. doi: 10.1371/journal.pone.0157461 (PMC4911011; doi:10.1371/journal.pone.0157461)
Supplement: S1 Fig — (DOCX) [file pone.0157461.s002.docx]

Records identified through database searching: (until May 2016)
PUBMED (n=3526)

EMBASE (n= 3046)

Web of Science (3851)

n= 10423

Additional records identified through hand search
(n =1)

Records screened after duplicates removed
(n =5083)

Records excluded: duplicates, title, abstract, not relevant, case-report, mechanism study

(n=5033)

Full-text articles assessed for eligibility
(n = 47 studies; 50 reports)

Full-text articles excluded, with reasons (n =23)

Reported only association between dairy protein and adiposity measure (n=1)

Cross-sectional study (n=11)

Years of age <18 (n=7)

Cluster analysis (n=1)

Review (n=3)

Studies included in qualitative synthesis
(n =24 studies; 27 reports)

One full-text articles excluded because:

Drapeu et al. 2004 (report no quantitative amount of dairy intake); Kaikkonen et al. 2015 (report no standard error or standard deviation)

Studies included in quantitative synthesis (meta-analysis)

n= 22 longitudinal studies

(25 reports):
