# Supplementary material for: Consumption of Dairy Products in Relation to Changes in Anthropometric Variables in Adult Populations: A Systematic Review and Meta-Analysis of Cohort Studies
Source: PLoS One. 2016 Jun 16;11(6):e0157461. doi: 10.1371/journal.pone.0157461 (PMC4911011; doi:10.1371/journal.pone.0157461)
Supplement: S1 Table — (DOCX) [file pone.0157461.s008.docx]

Full-text articles excluded with reasons:

| **Reference** | **Reason for exclusion** |
| --- | --- |
| Lee H-J, Cho J-i, Lee H-SH, Kim C-i, Cho E. Intakes of Dairy Products and Calcium and Obesity in Korean Adults: Korean National Health and Nutrition Examination Surveys (KNHANES) 2007-2009. PLoS ONE 2014, 9(6): e99085. | Cross-sectional study |
| Mirmiran P, Esmaillzadeh A & Azizi F. Dairy consumption and body mass index: an inverse relationship. International Journal of Obesity (Lond) 2005, 29: 115–121 | Cross-sectional study |
| Varenna M, Binelli L, Casari S, et al. (2007) Effects of dietary calcium intake on body weight and prevalence of osteoporosis in early postmenopausal women. American Journal of Clinical Nutrition 86, 639–644. | Cross-sectional study |
| Murakami K, Okubo H & Sasaki S. No relation between intakes of calcium and dairy products and body mass index in Japanese women aged 18 to 20 y. Nutrition 2006, 22: 490–495. | Cross-sectional study |
| Azadbakht L & Esmaillzadeh A. Dietary and nondietary determinants of central adiposity among Tehrani women. Public Health Nutr 2008, 11: 528–534. | Cross-sectional study |
| Snijder MB, van der Heijden A, van Dam RM, et al. Is higher dairy consumption associated with lower body weight and fewer metabolic disturbances? The Hoorn Study. The American journal of clinical nutrition 2007, 85: 989–995 | Cross-sectional study |
| Brooks BM, Rajeshwari R, Nicklas TA, et al. Association of calcium intake, dairy product consumption with overweight status in young adults (1995–1996): the Bogalusa Heart Study. Journal American College of Nutrition 2006, 25: 523–532. | Cross-sectional study |
| Beydoun MA, Gary TL, Caballero BH, et al. Ethnic differences in dairy and related nutrient consumption among US adults and their association with obesity, central obesity, and the metabolic syndrome. The American journal of clinical nutrition 2008, 87: 1914–1925. | Cross-sectional study |
| Marques-Vidal P, Goncalves A & Dias CM. Milk intake is inversely related to obesity in men and in young women: data from the Portuguese Health Interview Survey  1998–1999. International Journal of Obesity 2006, 30: 88–93 | Cross-sectional study |
| Lawlor DA, Ebrahim S, Timpson N, et al. Avoiding milk is associated with a reduced risk of insulin resistance and the metabolic syndrome: findings from the British Women’s Heart and Health Study. Diabetic Medicine 2005, 22: 808–811 | Cross-sectional study |
| Rosell M, Johansson G, Berglund L, et al. (2004) Associations between the intake of dairy fat and calcium and abdominal obesity. International Journal Obesity Related Metabolic Disorders 2004, 28: 1427–1434 | Cross-sectional study |
| [Berkey CS](http://www.ncbi.nlm.nih.gov/pubmed?term=Berkey%20CS%5BAuthor%5D&cauthor=true&cauthor_uid=15939853), [Rockett HR](http://www.ncbi.nlm.nih.gov/pubmed?term=Rockett%20HR%5BAuthor%5D&cauthor=true&cauthor_uid=15939853), [Willett WC](http://www.ncbi.nlm.nih.gov/pubmed?term=Willett%20WC%5BAuthor%5D&cauthor=true&cauthor_uid=15939853), [Colditz GA](http://www.ncbi.nlm.nih.gov/pubmed?term=Colditz%20GA%5BAuthor%5D&cauthor=true&cauthor_uid=15939853). Milk, dairy fat, dietary calcium, and weight gain: a longitudinal study of adolescents. [Archives of Pediatric Adolescent Medicine](http://www.ncbi.nlm.nih.gov/pubmed/15939853) 2005;159:543-50. | <18 years of age |
| [Berz JP](http://www.ncbi.nlm.nih.gov/pubmed?term=Berz%20JP%5BAuthor%5D&cauthor=true&cauthor_uid=21646587), [Singer MR](http://www.ncbi.nlm.nih.gov/pubmed?term=Singer%20MR%5BAuthor%5D&cauthor=true&cauthor_uid=21646587), [Guo X](http://www.ncbi.nlm.nih.gov/pubmed?term=Guo%20X%5BAuthor%5D&cauthor=true&cauthor_uid=21646587), [Daniels SR](http://www.ncbi.nlm.nih.gov/pubmed?term=Daniels%20SR%5BAuthor%5D&cauthor=true&cauthor_uid=21646587), [Moore LL](http://www.ncbi.nlm.nih.gov/pubmed?term=Moore%20LL%5BAuthor%5D&cauthor=true&cauthor_uid=21646587). Use of a DASH food group score to predict excess weight gain in adolescent girls in the National Growth and Health Study. [[Archives of Pediatric Adolescent Medicine](http://www.ncbi.nlm.nih.gov/pubmed/15939853)](http://www.ncbi.nlm.nih.gov/pubmed/21646587) 2011; 165:540-6. | <18 years of age |
| [Huh SY](http://www.ncbi.nlm.nih.gov/pubmed?term=Huh%20SY%5BAuthor%5D&cauthor=true&cauthor_uid=20338282), [Rifas-Shiman SL](http://www.ncbi.nlm.nih.gov/pubmed?term=Rifas-Shiman%20SL%5BAuthor%5D&cauthor=true&cauthor_uid=20338282), [Rich-Edwards JW](http://www.ncbi.nlm.nih.gov/pubmed?term=Rich-Edwards%20JW%5BAuthor%5D&cauthor=true&cauthor_uid=20338282), [Taveras EM](http://www.ncbi.nlm.nih.gov/pubmed?term=Taveras%20EM%5BAuthor%5D&cauthor=true&cauthor_uid=20338282), [Gillman MW](http://www.ncbi.nlm.nih.gov/pubmed?term=Gillman%20MW%5BAuthor%5D&cauthor=true&cauthor_uid=20338282). Prospective association between milk intake and adiposity in preschool-aged children. [Journal American Dietetic Association](http://www.ncbi.nlm.nih.gov/pubmed/20338282) 2010;110:563-70. | <18 years of age |
| [Lin SL](http://www.ncbi.nlm.nih.gov/pubmed?term=Lin%20SL%5BAuthor%5D&cauthor=true&cauthor_uid=23285099), [Tarrant M](http://www.ncbi.nlm.nih.gov/pubmed?term=Tarrant%20M%5BAuthor%5D&cauthor=true&cauthor_uid=23285099), [Hui LL](http://www.ncbi.nlm.nih.gov/pubmed?term=Hui%20LL%5BAuthor%5D&cauthor=true&cauthor_uid=23285099), [Kwok MK](http://www.ncbi.nlm.nih.gov/pubmed?term=Kwok%20MK%5BAuthor%5D&cauthor=true&cauthor_uid=23285099), [Lam TH](http://www.ncbi.nlm.nih.gov/pubmed?term=Lam%20TH%5BAuthor%5D&cauthor=true&cauthor_uid=23285099), [Leung GM](http://www.ncbi.nlm.nih.gov/pubmed?term=Leung%20GM%5BAuthor%5D&cauthor=true&cauthor_uid=23285099), [Schooling CM](http://www.ncbi.nlm.nih.gov/pubmed?term=Schooling%20CM%5BAuthor%5D&cauthor=true&cauthor_uid=23285099). The role of dairy products and milk in adolescent obesity: evidence from Hong Kong's "Children of 1997" birth cohort. [PLoS One.](http://www.ncbi.nlm.nih.gov/pubmed/?term=The+role+of+dairy+products+and+milk+in+adolescent+obesity%3A+evidence+from+Hong+Kong%27s+%22Children+of+1997%22+birth+cohort) 2012;7:e52575. | <18 years of age |
| [Phillips SM](http://www.ncbi.nlm.nih.gov/pubmed?term=Phillips%20SM%5BAuthor%5D&cauthor=true&cauthor_uid=12917718), [Bandini LG](http://www.ncbi.nlm.nih.gov/pubmed?term=Bandini%20LG%5BAuthor%5D&cauthor=true&cauthor_uid=12917718), [Cyr H](http://www.ncbi.nlm.nih.gov/pubmed?term=Cyr%20H%5BAuthor%5D&cauthor=true&cauthor_uid=12917718), [Colclough-Douglas S](http://www.ncbi.nlm.nih.gov/pubmed?term=Colclough-Douglas%20S%5BAuthor%5D&cauthor=true&cauthor_uid=12917718), [Naumova E](http://www.ncbi.nlm.nih.gov/pubmed?term=Naumova%20E%5BAuthor%5D&cauthor=true&cauthor_uid=12917718), [Must A](http://www.ncbi.nlm.nih.gov/pubmed?term=Must%20A%5BAuthor%5D&cauthor=true&cauthor_uid=12917718). Dairy food consumption and body weight and fatness studied longitudinally over the adolescent period.  [International Journal Obesity Related Metabolic Disorders.](http://www.ncbi.nlm.nih.gov/pubmed/12917718) 2003 Sep;27(9):1106-13. | <18 years of age |
| [Scharf RJ](http://www.ncbi.nlm.nih.gov/pubmed?term=Scharf%20RJ%5BAuthor%5D&cauthor=true&cauthor_uid=23508869), [Demmer RT](http://www.ncbi.nlm.nih.gov/pubmed?term=Demmer%20RT%5BAuthor%5D&cauthor=true&cauthor_uid=23508869), [DeBoer MD](http://www.ncbi.nlm.nih.gov/pubmed?term=DeBoer%20MD%5BAuthor%5D&cauthor=true&cauthor_uid=23508869). Longitudinal evaluation of milk type consumed and weight status in preschoolers. [Archives of disease in childhood.](http://www.ncbi.nlm.nih.gov/pubmed/?term=Longitudinal+evaluation+of+milk+type+consumed+and+weight+status+in+preschoolers) 2013;98(5):335-40. | <18 years of age |
| [te Velde SJ](http://www.ncbi.nlm.nih.gov/pubmed?term=te%20Velde%20SJ%5BAuthor%5D&cauthor=true&cauthor_uid=21332840), [Snijder MB](http://www.ncbi.nlm.nih.gov/pubmed?term=Snijder%20MB%5BAuthor%5D&cauthor=true&cauthor_uid=21332840), [van Dijk AE](http://www.ncbi.nlm.nih.gov/pubmed?term=van%20Dijk%20AE%5BAuthor%5D&cauthor=true&cauthor_uid=21332840), [Brug J](http://www.ncbi.nlm.nih.gov/pubmed?term=Brug%20J%5BAuthor%5D&cauthor=true&cauthor_uid=21332840), [Koppes LL](http://www.ncbi.nlm.nih.gov/pubmed?term=Koppes%20LL%5BAuthor%5D&cauthor=true&cauthor_uid=21332840), [van Mechelen W](http://www.ncbi.nlm.nih.gov/pubmed?term=van%20Mechelen%20W%5BAuthor%5D&cauthor=true&cauthor_uid=21332840), [Twisk JW](http://www.ncbi.nlm.nih.gov/pubmed?term=Twisk%20JW%5BAuthor%5D&cauthor=true&cauthor_uid=21332840). Dairy intake from adolescence into adulthood is not associated with being overweight and metabolic syndrome in adulthood: the Amsterdam Growth and Health Longitudinal Study.  [Journal of Human Nutrition and Dietetics](http://www.ncbi.nlm.nih.gov/pubmed/21332840) 2011;24:233-44. | <18 years of age |
| [Astrup A](http://www.ncbi.nlm.nih.gov/pubmed?term=Astrup%20A%5BAuthor%5D&cauthor=true&cauthor_uid=24695891). Yogurt and dairy product consumption to prevent cardiometabolic diseases: epidemiologic and experimental studies. The American journal of clinical nutrition 2014;99:1235S-42S. | Review |
| [Dougkas A](http://www.ncbi.nlm.nih.gov/pubmed?term=Dougkas%20A%5BAuthor%5D&cauthor=true&cauthor_uid=21320381), [Reynolds CK](http://www.ncbi.nlm.nih.gov/pubmed?term=Reynolds%20CK%5BAuthor%5D&cauthor=true&cauthor_uid=21320381), [Givens ID](http://www.ncbi.nlm.nih.gov/pubmed?term=Givens%20ID%5BAuthor%5D&cauthor=true&cauthor_uid=21320381), [Elwood PC](http://www.ncbi.nlm.nih.gov/pubmed?term=Elwood%20PC%5BAuthor%5D&cauthor=true&cauthor_uid=21320381), [Minihane AM](http://www.ncbi.nlm.nih.gov/pubmed?term=Minihane%20AM%5BAuthor%5D&cauthor=true&cauthor_uid=21320381). Associations between dairy consumption and body weight: a review of the evidence and underlying mechanisms.  [Nutrition Research Reviwes.](http://www.ncbi.nlm.nih.gov/pubmed/21320381) 2011;24:72-95. | Review |
| [Kratz M](http://www.ncbi.nlm.nih.gov/pubmed?term=Kratz%20M%5BAuthor%5D&cauthor=true&cauthor_uid=22810464), [Baars T](http://www.ncbi.nlm.nih.gov/pubmed?term=Baars%20T%5BAuthor%5D&cauthor=true&cauthor_uid=22810464), [Guyenet S](http://www.ncbi.nlm.nih.gov/pubmed?term=Guyenet%20S%5BAuthor%5D&cauthor=true&cauthor_uid=22810464). The relationship between high-fat dairy consumption and obesity, cardiovascular, and metabolic disease. [European Journal of Nutrition](http://www.ncbi.nlm.nih.gov/pubmed/22810464) 2013;52:1-24. | Review |
| [Louie JC](http://www.ncbi.nlm.nih.gov/pubmed?term=Louie%20JC%5BAuthor%5D&cauthor=true&cauthor_uid=21521450), [Flood VM](http://www.ncbi.nlm.nih.gov/pubmed?term=Flood%20VM%5BAuthor%5D&cauthor=true&cauthor_uid=21521450), [Hector DJ](http://www.ncbi.nlm.nih.gov/pubmed?term=Hector%20DJ%5BAuthor%5D&cauthor=true&cauthor_uid=21521450), [Rangan AM](http://www.ncbi.nlm.nih.gov/pubmed?term=Rangan%20AM%5BAuthor%5D&cauthor=true&cauthor_uid=21521450), [Gill TP](http://www.ncbi.nlm.nih.gov/pubmed?term=Gill%20TP%5BAuthor%5D&cauthor=true&cauthor_uid=21521450). Dairy consumption and overweight and obesity: a systematic review of prospective cohort studies. [Obesity Rev](http://www.ncbi.nlm.nih.gov/pubmed/21521450)iews 2011;12:e582-92. | Review |
| Newby PK, Muller D, Hallfrisch J, Qiao N, Andres R, Tucker KL. Dietary patterns and changes in body mass index and waist circumference in adults. The American journal of clinical nutrition 2003, 77: 1417-1425. | Cluster analysis |
| [Halkjær J](http://www.ncbi.nlm.nih.gov/pubmed?term=Halkj%C3%A6r%20J%5BAuthor%5D&cauthor=true&cauthor_uid=21139559), [Olsen A](http://www.ncbi.nlm.nih.gov/pubmed?term=Olsen%20A%5BAuthor%5D&cauthor=true&cauthor_uid=21139559), [Overvad K](http://www.ncbi.nlm.nih.gov/pubmed?term=Overvad%20K%5BAuthor%5D&cauthor=true&cauthor_uid=21139559), [Jakobsen MU](http://www.ncbi.nlm.nih.gov/pubmed?term=Jakobsen%20MU%5BAuthor%5D&cauthor=true&cauthor_uid=21139559), [Boeing H](http://www.ncbi.nlm.nih.gov/pubmed?term=Boeing%20H%5BAuthor%5D&cauthor=true&cauthor_uid=21139559), [Buijsse B](http://www.ncbi.nlm.nih.gov/pubmed?term=Buijsse%20B%5BAuthor%5D&cauthor=true&cauthor_uid=21139559), [Palli D](http://www.ncbi.nlm.nih.gov/pubmed?term=Palli%20D%5BAuthor%5D&cauthor=true&cauthor_uid=21139559), [Tognon G](http://www.ncbi.nlm.nih.gov/pubmed?term=Tognon%20G%5BAuthor%5D&cauthor=true&cauthor_uid=21139559), [Du H](http://www.ncbi.nlm.nih.gov/pubmed?term=Du%20H%5BAuthor%5D&cauthor=true&cauthor_uid=21139559), [van der A DL](http://www.ncbi.nlm.nih.gov/pubmed?term=van%20der%20A%20DL%5BAuthor%5D&cauthor=true&cauthor_uid=21139559), [Forouhi NG](http://www.ncbi.nlm.nih.gov/pubmed?term=Forouhi%20NG%5BAuthor%5D&cauthor=true&cauthor_uid=21139559), [Wareham NJ](http://www.ncbi.nlm.nih.gov/pubmed?term=Wareham%20NJ%5BAuthor%5D&cauthor=true&cauthor_uid=21139559), [Feskens EJ](http://www.ncbi.nlm.nih.gov/pubmed?term=Feskens%20EJ%5BAuthor%5D&cauthor=true&cauthor_uid=21139559), [Sørensen TI](http://www.ncbi.nlm.nih.gov/pubmed?term=S%C3%B8rensen%20TI%5BAuthor%5D&cauthor=true&cauthor_uid=21139559), [Tjønneland A](http://www.ncbi.nlm.nih.gov/pubmed?term=Tj%C3%B8nneland%20A%5BAuthor%5D&cauthor=true&cauthor_uid=21139559). Intake of total, animal and plant protein and subsequent changes in weight or waist circumference in European men and women: the Diogenes project. [International Journal of Obesity (Lond).](http://www.ncbi.nlm.nih.gov/pubmed/21139559) 2011 Aug;35:1104-13 | Reported only association between dairy protein and adiposity measure |
